# Endoglin Protein Interactome Profiling Identifies TRIM21 and Galectin-3 as New Binding Partners

**DOI:** 10.3390/cells8091082

**Published:** 2019-09-13

**Authors:** Eunate Gallardo-Vara, Lidia Ruiz-Llorente, Juan Casado-Vela, María J. Ruiz-Rodríguez, Natalia López-Andrés, Asit K. Pattnaik, Miguel Quintanilla, Carmelo Bernabeu

**Affiliations:** 1 Centro de Investigaciones Biológicas, Consejo Superior de Investigaciones Científicas (CSIC), and Centro de Investigación Biomédica en Red de Enfermedades Raras (CIBERER), 28040 Madrid, Spain; eunate.gallardo@yale.edu (E.G.-V.); lruiz.llorente@cib.csic.es (L.R.-L.); 2 Bioengineering and Aerospace Engineering Department, Universidad Carlos III and Centro de Investigación Biomédica en Red Enfermedades Neurodegenerativas (CIBERNED), Leganés, 28911 Madrid, Spain; jucasado@ing.uc3m.es; 3 Centro Nacional de Investigaciones Cardiovasculares (CNIC), 28029 Madrid, Spain; mariajesus.ruiz@cnic.es; 4 Cardiovascular Translational Research, Navarrabiomed, Complejo Hospitalario de Navarra (CHN), Universidad Pública de Navarra (UPNA), IdiSNA, 31008 Pamplona, Spain; natalia.lopez.andres@navarra.es; 5 School of Veterinary Medicine and Biomedical Sciences, and Nebraska Center for Virology, University of Nebraska-Lincoln, Lincoln, NE 68583, USA; apattnaik2@unl.edu; 6 Instituto de Investigaciones Biomédicas “Alberto Sols”, Consejo Superior de Investigaciones Científicas (CSIC), and Departamento de Bioquímica, Universidad Autónoma de Madrid (UAM), 28029 Madrid, Spain

**Keywords:** endothelial cells, endoglin, TGF-β, BMP, HHT, cancer, preeclampsia, TRIM family, galectin family, protein–protein interactions

## Abstract

Endoglin is a 180-kDa glycoprotein receptor primarily expressed by the vascular endothelium and involved in cardiovascular disease and cancer. Heterozygous mutations in the endoglin gene (ENG) cause hereditary hemorrhagic telangiectasia type 1, a vascular disease that presents with nasal and gastrointestinal bleeding, skin and mucosa telangiectases, and arteriovenous malformations in internal organs. A circulating form of endoglin (alias soluble endoglin, sEng), proteolytically released from the membrane-bound protein, has been observed in several inflammation-related pathological conditions and appears to contribute to endothelial dysfunction and cancer development through unknown mechanisms. Membrane-bound endoglin is an auxiliary component of the TGF-β receptor complex and the extracellular region of endoglin has been shown to interact with types I and II TGF-β receptors, as well as with BMP9 and BMP10 ligands, both members of the TGF-β family. To search for novel protein interactors, we screened a microarray containing over 9000 unique human proteins using recombinant sEng as bait. We find that sEng binds with high affinity, at least, to 22 new proteins. Among these, we validated the interaction of endoglin with galectin-3, a secreted member of the lectin family with capacity to bind membrane glycoproteins, and with tripartite motif-containing protein 21 (TRIM21), an E3 ubiquitin-protein ligase. Using human endothelial cells and Chinese hamster ovary cells, we showed that endoglin co-immunoprecipitates and co-localizes with galectin-3 or TRIM21. These results open new research avenues on endoglin function and regulation.

## 1. Introduction

Endoglin is an auxiliary TGF-β co-receptor predominantly expressed in endothelial cells, which is involved in vascular development, repair, homeostasis, and disease [1,2,3,4]. Heterozygous mutations in the human ENDOGLIN gene (ENG) cause hereditary hemorrhagic telangiectasia (HHT) type 1, a vascular disease associated with nasal and gastrointestinal bleeds, telangiectases on skin and mucosa and arteriovenous malformations in the lung, liver, and brain [4,5,6]. The key role of endoglin in the vasculature is also illustrated by the fact that endoglin-KO mice die in utero due to defects in the vascular system [7]. Endoglin expression is markedly upregulated in proliferating endothelial cells involved in active angiogenesis, including the solid tumor neovasculature [8,9]. For this reason, endoglin has become a promising target for the antiangiogenic treatment of cancer [10,11,12]. Endoglin is also expressed in cancer cells where it can behave as both a tumor suppressor in prostate, breast, esophageal, and skin carcinomas [13,14,15,16] and a promoter of malignancy in melanoma and Ewing’s sarcoma [17]. Ectodomain shedding of membrane-bound endoglin may lead to a circulating form of the protein, also known as soluble endoglin (sEng) [18,19,20]. Increased levels of sEng have been found in several vascular-related pathologies, including preeclampsia, a disease of high prevalence in pregnant women which, if left untreated, can lead to serious and even fatal complications for both mother and baby [2,18,19,21]. Interestingly, several lines of evidence support a pathogenic role of sEng in the vascular system, including endothelial dysfunction, antiangiogenic activity, increased vascular permeability, inflammation-associated leukocyte adhesion and transmigration, and hypertension [18,22,23,24,25,26,27]. Because of its key role in vascular pathology, a large number of studies have addressed the structure and function of endoglin at the molecular level, in order to better understand its mechanism of action.

Endoglin is a 180-kDa disulphide-linked homodimeric type I transmembrane glycoprotein that contains a large extracellular region (561 amino acids), a single hydrophobic transmembrane domain, and a short (47 amino acids) cytosolic tail [28,29]. As the extracellular region of endoglin comprises most (~88%) of the whole protein and is present in circulating sEng, which displays key relevant functions in vascular biology, several reports have analyzed its structure and interacting protein partners. Studies of the three-dimensional structure of the ectodomain of endoglin, using single particle electron microscopy [30], as well as X-ray crystallography [31] have revealed the presence of an N-terminal orphan region (OR), and a C-terminal bipartite zona pellucida (ZP) module. The conserved ZP module of endoglin is likely involved in polymerization of extracellular proteins, as reported for several ZP protein family members [31,32]. In addition, the endoglin ZP module contains an accessible arginine–glycine aspartic acid (RGD) sequence located in the ZP–N subdomain, which is a consensus binding motif for integrin recognition [28,31]. Notably, the RGD motif of membrane-bound and soluble endoglin appears to be actively involved in integrin-mediated cell adhesion, implicating, at least, α5β1 and αvβ3 integrin family members [24,25,33]. The orphan domain of endoglin is responsible for the binding with high affinity to members of the TGF-β family, namely BMP9 and BMP10 [31,34,35]. Furthermore, in line with the initially recognized role of endoglin as an essential co-receptor in TGF-β family signaling [4,36,37,38,39], the extracellular region of endoglin can simultaneously bind to BMP9 and the endothelial TGF-β type I receptor ALK1 [31,34,40]. Moreover, the ectodomain of endoglin specifically interacts with the TGF-β type I receptor ALK5 and with the TGF-β type II receptor [41,42]. Of note, the matrix metalloprotease 14 (MMP14 or MT1-MMP) can bind with and target the extracellular region of membrane-bound endoglin to release sEng (either alone or in complex with exosomes) upon proteolytic processing [22,23,42,43]. In addition, in the cancer setting, it has been shown that sEng associates with the receptor tyrosine kinase Met and antagonizes Met signaling in spindle carcinoma cells [44]. Nevertheless, the current knowledge about protein interactors of the endoglin ectodomain does not fully explain the exact molecular mechanisms of action involved in the complex cellular and pathophysiological roles of endoglin.

In this study, we screened a microarray containing over 9000 unique human proteins using recombinant sEng as bait. Among the 22 new putative high-affinity interactors identified, we demonstrated for the first time the specific interaction of endoglin with galectin-3, a secreted member of the lectin family with capacity to bind membrane glycoproteins, and with tripartite motif-containing protein 21 (TRIM21), an E3 ubiquitin-protein ligase. Taken together, this proteomic study reveals novel avenues on endoglin function and regulation.

## 2. Materials and Methods

### 2.1. Cell-free Protein Microarrays

Protein interaction screening was carried out using the ProtoArray human protein Microarray v5.0 (Life Technologies), based on the nucleic acid programmable protein arrays (NAPPA) technology [45]. This microarray contains over 9000 unique human proteins individually purified and arrayed under native conditions to maximize functionality. Two replicate analyses were hybridized with purified recombinant human soluble endoglin (sEng; Glu26-Gly586; 1097-EN, R&D Systems). Arrays were then incubated with a mouse monoclonal antibody (mAb) against human endoglin (clone SN6h, Dako) and, after washing, with a rabbit antimouse secondary antibody coupled to horseradish peroxidase [HRP] (P0260, Dako). The specific binding signal was developed using SuperSignalTM West Pico Plus chemiluminescent substrate (34580, Thermo Fisher Scientific). Arrays were scanned using an Axon GenePix 4100A microarray reader with the GenePixPro 6.0 program and results analyzed with Prospector software (Invitrogen). After scanning, chemoluminescence in specific regions on the surface of arrays were measured and statistically analyzed using Z-scores [46], with the formula: Z = (x − µ)/σ, where x is the raw value, µ the population mean, and σ the SD for the population. Only those proteins reaching a Z-score ≥2.0 in two independent protein arrays were considered as candidate bona fide interacting proteins for further validation analyses.

### 2.2. Cell Culture

All cell types were incubated routinely at 37 °C in a humidified atmosphere with 5% CO2. Human umbilical vein-derived endothelial cells (HUVECs) were purchased from Lonza and used at early passages (3–5). HUVECs were grown on 0.2% gelatin (Sigma-Aldrich) pre-coated plates in endothelial basal medium (EBM2) supplemented with EGM2 SingleQuots (EBM2/EGM2 medium; Lonza). The Chinese hamster ovary cell line CHO-K1 was cultured in Dulbecco’s modified Eagle’s medium/Nutrient mixture F-12 medium (DMEM:F12). All cell media were supplemented with 10% heat-inactivated fetal bovine serum (FBS, Gibco), 2 mM L-glutamine, 100 U/mL penicillin, and 100 µg/mL streptomycin (Gibco), unless otherwise noted.

### 2.3. Plasmids and Cell Transfections

The following human endoglin expression vectors were used: pDisplay–HA–EngFL (full-length), pcEXV–HA–EngFL (full-length), pDisplay–HA–EngEC (extracellular region), pDisplay–HA–EngTMEC (transmembrane and extracellular regions), pDisplay–HA–Mock (empty vector; Ø) and pcEXV–Ø (empty vector; Ø), all of them previously described [25,37,41]. Some of these vectors encode endoglin constructs tagged with the human influenza hemagglutinin (HA) epitope, as indicated. Human TRIM21 or human galectin-3 were overexpressed using the pcDNA3.1–HA–hTRIM21 [47] or pcDNA3.1–Gal-3 [48], respectively. All plasmids were confirmed by sequencing (Secugen, Madrid). Transient transfections of CHO-K1 cells with plasmids were carried out using Lipofectamine 2000 (Invitrogen). Cells were analyzed after 48 h of transfection, for protein expression by Western blot.

### 2.4. Immunoprecipitation Assays

HUVECs or CHO-K1 cells were lysed in lysis buffer (1% NP-40, 0.5 mM NaF, 150 mM NaCl, 50 mM Tris/pH 7.4, and 1 mM phenylmethylsulfonyl fluoride (PMSF)), supplemented with protease (Roche) and phosphatase (Calbiochem) inhibitors. After 5 min of incubation at 4 °C, lysates were centrifuged at 10,000∙*g* for 5 min and precleared using Protein G magnetic beads (PureProteome-Protein G magnetic beads, Millipore). Protein concentration of whole-cell extracts was measured using the Bradford quantification method (Bio-Rad protein Assay) in a Novaspek Plus Visible Spectrophotometer (GE Healthcare Life Sciences).

Immunoprecipitations (IPs) for Western blot analysis were carried out using Protein G magnetic beads incubated with the indicated primary antibodies. For galectin-3/endoglin IP, mouse mAb anti-galectin-3 (IgG1, clone B2C10, sc-32790, Santa Cruz Biotech) and mouse mAb anti-HA (IgG1, clone CB051, #TA180128, Origin) were used. For TRIM21/endoglin IP, rabbit mAb anti-TRIM21 (#92043, Cell Signaling Technology) and mouse mAb anti-endoglin (P4A4, sc-20072, Santa Cruz Biotech) were used. In all cases, control immunoprecipitations with isotype-matched antibodies (Immunostep) were carried out. Antibodies were incubated with protein G magnetic beads for 10 min at room temperature, followed by several washes with PBS. Then, antibody-coupled protein G magnetic beads were incubated with total cell lysates (~0.5 mg) overnight at 4 °C. After washing with PBS, immunoprecipitates were further analyzed for Western blot analysis.

Co-IPs for proteomic analysis (mass spectrometry) were carried out by incubation of 1 mg of protein lysates with protein G-coated magnetic beads coupled with either the monoclonal antibody P4A4 anti-endoglin (Developmental Studies Hybridoma Bank, The University of Iowa, Iowa City, IA, USA) or an isotype-matched (IgG2b) control antibody (Immunostep, Salamanca, Spain). An additional control with protein G magnetic beads in the absence of antibodies was also included. After extensive washing with PBS, immunoprecipitates were then subjected to mass spectrometry analysis.

### 2.5. Mass Spectrometry and Data Analysis

Samples from co-IPs were incubated overnight at 4 °C on a rotator, washed twice and then incubated with Laemmli buffer at 95ºC for 5 min. Proteins eluted from each condition (P4A4, IgG2b, protein G) were analyzed by SDS-PAGE (10% polyacrilamide and 0.1% sodium dodecyl sulphate under nonreducing and albumin-free conditions, and then stained with colloidal Coomassie Brilliant Blue (G-250, Sigma). Each lane of gel was divided into small sections, followed by a standard digestion protocol with trypsin [49,50]. Peptides were trapped onto a C18-A1 ASY-Column (2 cm, ID100 μm, 5μm) (Thermo Fisher Scientific), and then eluted onto a Biosphere C18 column (75 μm, 16 cm, 3 μm) (NanoSeparations) and separated using a 110 min gradient min (90 min 0–35% Buffer B, 10 min 35–45% Buffer B, 4 min 45–95% Buffer B, 5 min 95% Buffer B, and 1 min 0% Buffer B) (Buffer A: 0.1% formic acid, 2% acetonitrile; Buffer B: 0.1% formic acid in acetonitrile) at a flow-rate of 200 nL/min on a nanoEasy HPLC (Proxeon) coupled to a nanoelectrospray (Thermo Fisher Scientific). Mass spectra were acquired on an LTQ-Orbitrap Velos mass spectrometer (Thermo Fisher Scientific) in the positive ion mode. Full-scan MS spectra (m/z 400–2000) were acquired in the Orbitrap at a resolution of 60,000, and the 15 most intense ions were selected for collision-induced dissociation (CID) fragmentation in the linear ion trap with a normalized collision energy of 35%. Singly charged ions and unassigned charge states were rejected. Dynamic exclusion was enabled with an exclusion duration of 30 s. Mass spectra *.raw files were searched against the human SwissProt 2016_10 database (20,121 sequence protein entries) using the MASCOT search engine (version 2.3, Matrix Science. Precursor and fragment mass tolerance were set to 10 ppm and 0.5 Da, respectively, allowing 2 missed cleavages, N-terminal, carbamidomethylation of cysteines as a fixed modification and methionine oxidation as a variable modification. Identified peptides were validated using Percolator algorithm with a q-value threshold of 0.01 [51]. Peptides found in the analysis, which covered ≥2% of a specific protein region, allowed identifying the proteins present in the sample. These proteins were then subjected to String-protein database version 11 to analyze possible interactions already described, between them. The protein identification by nLC–MS/MS was carried out by the Proteomics and Genomics Facility of Biological Research Center (Centro de Investigaciones Biológicas; CIB-CSIC), a member of the ProteoRed-ISCIII network. 

### 2.6. Western Blot Analysis

Total cell lysates or immunoprecipitates were separated by SDS-PAGE on 10% acrylamide gels under reducing or nonreducing conditions, and resolved proteins were electrotransferred to polyvinylidene difluoride (PVDF) or nitrocellulose membranes using the semidry system iBlot Gel transfer (Invitrogen). Then, membranes were blocked with TBS buffer (0.1% Tween, 5% BSA) for 1 h at room temperature and immunoblotted with primary antibodies, as indicated. For galectin-3/endoglin IP, anti-galectin-3 (biotinylated polyclonal goat IgG; BAF1154, R&D Systems) and anti-endoglin (rabbit mAb, clone EPR10145-12, Abcam), antibodies were used. For TRIM21/endoglin IP, anti-TRIM21 (rabbit mAb #92043, Cell Signaling Technology), or anti-HA (mouse mAb, clone CB051, #TA180128, OriGene) antibodies were used. Loading control antibodies were also included (anti-β-actin; A-1978, Sigma). Incubation with the primary antibodies was carried out overnight at 4 °C. The next day, membranes were washed and incubated in TBS with the specific secondary antibodies coupled to HRP, rabbit anti-mouse (1:1000 dilution; P0260, Dako) or goat anti-rabbit (1:1000 dilution; P0448, Dako), or with streptavidin-HRP conjugate (1.5 µg/mL; SA10001, Thermo Fisher Scientific). Protein bands were revealed using SuperSignalTM West Pico Plus chemiluminescent substrate (34580, Thermo Fisher Scientific) to enhance signal, following the manufacturer’s protocol. Bands were visualized and quantified using the Molecular Imager® Gel DocTM XR+ System with Image Lab software (Bio-Rad).

### 2.7. Immunofluorescence Microscopy

HUVECs were grown to confluence in 12 mm diameter coverslips, previously coated with 0.2% gelatin (Sigma-Aldrich) in PBS. Cells were fixed with 3% paraformaldehyde in PBS and permeabilized with 0.1% Triton X-100 during 10 min at room temperature. HUVECs were blocked with PBS containing either 2% BSA (galectin-3) or 1% BSA plus 5% normal goat serum [NGS] (TRIM21). In order to monitor the co-localization of endoglin with TRIM21 or galectin-3, samples were incubated first with the mouse monoclonal antibody anti-human endoglin (P4A4; sc-20072, Santa Cruz Biotech). After washing twice with PBS, samples were incubated with antibodies, anti-TRIM21 (rabbit mAb #92043, Cell Signaling Technology) or anti-galectin-3 (rabbit polyclonal IgG, #PA5-34819, Thermo Fisher Scientific). Then, after several washing steps with PBS, cells were incubated with the secondary antibodies Alexa 488 goat anti-mouse IgG or Alexa 647 goat anti-rabbit IgG. To visualize the samples and for nuclear staining, slides were mounted in Prolong-DAPI (4′,6-diamidino-2-phenylindole) reagent (Molecular Probes, Invitrogen). Samples were observed using a SP5 fluorescence confocal microscope (DMI6000 CS Leica Microsystems).

### 2.8. Bio-Layer Interferometry Measurements

Protein–protein interactions were measured by bio-layer interferometry using a single channel BLItz system (ForteBio BLItz®). Recombinant human endoglin/CD105 protein (1097-EN-025, R&D) was diluted to a final concentration of 250 µg/mL in PBS containing 0.1% BSA. Recombinant proteins tagged with 6xHis were resuspended following the manufacturer´s recommendations. Human full-length TRIM21 protein (RO52; 6His-tag, N-terminus, LSBio) was diluted to a final concentration of 250 µg/mL in 0.2 M Tris/pH 7.4 + 25% glycerol buffer. Human full-length galectin-3 protein tagged at the C-terminus (LGALS3; 6His-tag, LS-G39216, LSBio) or the N-terminus (LS-G26359; 6His-tag, LSBio) were diluted in distilled water or 10% glycerol in PBS/pH 7.4, respectively, to a final concentration of 100–200 µg/mL. The 6His-tagged proteins (galectin-3 or TRIM21) were immobilized on Ni–NTA (Nickel-nitrilotriacetic acid) biosensor tips and 4.1 µM soluble endoglin, or its corresponding control buffer was added to the biosensor to measure the endoglin-TRIM21 or endoglin-galectin-3 binding. Each binding reaction was carried out first with a 30 sec baseline (buffer), followed by a 300 s association phase (protein binding) and a 300 s dissociation phase (buffer only). Assays were performed by duplicate, and binding graphs were constructed by representing the experimental interferometry binding values (nm) and the duration of experiment run (sec). BLItz Pro software was used to analyze the data.

### 2.9. Statistical Analysis

All quantified results are shown as mean ± SD. Differences in mean values were analyzed using Student´s t test. Asterisks indicate statistically significant values between selected conditions (*p < 0.05; **p < 0.01; ***p < 0.005; ns not significant).

## 3. Results

### 3.1. Identification of Human Proteins Interacting with Endoglin Using Cell-Free Protein Microarray Technology

Recombinant sEng, encompassing the extracellular domain of human endoglin, was probed twice against a commercially available microarray that contains over 9000 unique human proteins individually purified and arrayed (Microarray v5.0, Life Technologies). Among these, we identified 22 proteins common to both replicates, strongly interacting with endoglin (Table 1). The stringent list of potential interactors was selected by imposing a Z-score ≥2.0 as a threshold.

Although the probe encoded the extracellular domain of endoglin, gene ontology analyses revealed that the selected proteins were located in different subcellular compartments, including the cytoplasm, nucleus, membrane, and extracellular subsets. Putative endoglin interactors included cytoskeletal components, ion channels, soluble factors, ubiquitin-ligases, RNA polymerases, extracellular matrix proteins, and membrane receptors, among others (Table 1). Among these, galectin-3 and TRIM21 were selected for validation due their involvement in several biological functions shared by endoglin.

### 3.2. Galectin-3 Interacts with Endoglin in Cells

**Figure 1:**
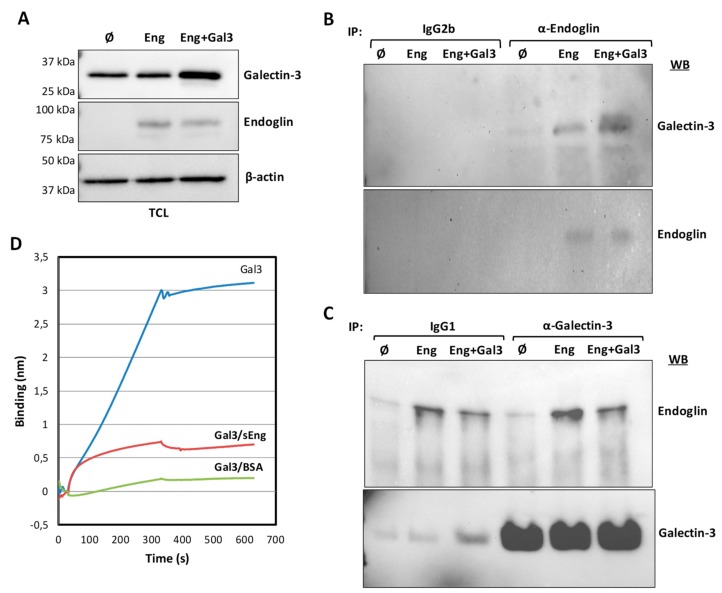
Protein–protein association between galectin-3 and endoglin. (**A**–**C**). Co-immunoprecipitation of galectin-3 and endoglin. CHO-K1 cells were transiently transfected with pcEXV-Ø (Ø), pcEXV–HA–EngFL (Eng) and pcDNA3.1–Gal-3 (Gal3) expression vectors. (**A**) Total cell lysates (TCL) were analyzed by SDS-PAGE under reducing conditions, followed by Western blot (WB) analysis using specific antibodies to endoglin, galectin-3 and β-actin (loading control). Cell lysates were subjected to immunoprecipitation (IP) with anti-endoglin (**B**) or anti-galectin-3 (**C**) antibodies, followed by SDS-PAGE under reducing conditions and WB analysis with anti-endoglin or anti-galectin-3 antibodies, as indicated. Negative controls with an IgG2b (**B**) and IgG1 (**C**) were included. (**D**) Protein-protein interactions between galectin-3 and endoglin using Bio-layer interferometry (BLItz). The Ni–NTA biosensors tips were loaded with 7.3 µM recombinant human galectin-3/6xHis at the C-terminus (LGALS3), and protein binding was measured against 0.1% BSA in PBS (negative control) or 4.1 µM soluble endoglin (sEng). Kinetic sensorgrams were obtained using a single channel ForteBioBLItzTM instrument.

Galectin-3 is a secreted member of the lectin family with the capacity to bind membrane glycoproteins like endoglin and is involved in the pathogenesis of many human diseases [52]. We confirmed the protein screen data for galectin-3, as evidenced by two-way co-immunoprecipitation of endoglin and galectin-3 upon co-transfection in CHO-K1 cells. As shown in Figure 1A, galectin-3 and endoglin were efficiently transfected, as demonstrated by Western blot analysis in total cell extracts. No background levels of endoglin were observed in control cells transfected with the empty vector (Ø). By contrast, galectin-3 could be detected in all samples but, as expected, showed an increased signal in cells transfected with the galectin-3 expression vector. Co-immunoprecipitation studies of these cell lysates showed that galectin-3 was present in endoglin immunoprecipitates (Figure 1B). Conversely, endoglin was also detected in galectin-3 immunoprecipitates (Figure 1C).

Next, we analyzed the interaction between galectin-3 and endoglin by biolayer interferometry [53,54] as implemented in the BLItzTM instrument, which allows kinetic analysis of protein–protein interactions using small amounts of protein. Galectin-3 with a 6His-tag at the C-terminus was immobilized on Ni–NTA biosensor tips, protein interactions were measured against soluble endoglin or its corresponding control buffer, and the association kinetics was monitored. As shown in Figure 1D, a specific interaction between endoglin and galectin-3 is visualized as compared to the background signal. Interestingly, similar experiments using galectin-3 with a 6His-tag at the N-terminus did not display any specific interaction with endoglin (data not shown), suggesting that the N-terminus of galectin-3 shelters the binding domain of endoglin.

**Figure 2:**
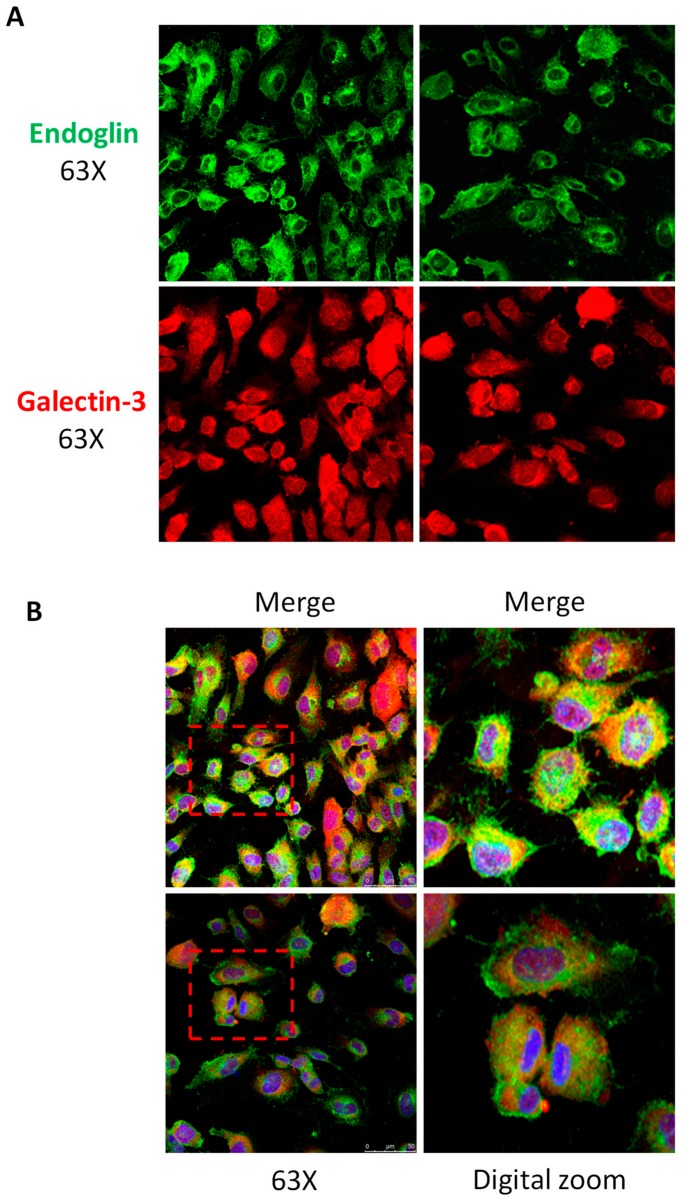
Galectin-3 and endoglin co-localize in human endothelial cells. Human umbilical vein-derived endothelial cell (HUVEC) monolayers were fixed with paraformaldehyde, permeabilized with Triton X-100, incubated with the mouse mAb P4A4 anti-endoglin, washed, and incubated with a rabbit polyclonal anti-galectin-3 antibody (PA5-34819). Galectin-3 and endoglin were detected by immunofluorescence upon incubation with Alexa 647 goat anti-rabbit IgG (red staining) and Alexa 488 goat anti-mouse IgG (green staining) secondary antibodies, respectively. (**A**) Single staining of galectin-3 (red) and endoglin (green) at the indicated magnifications. (**B**) Merge images plus DAPI (nuclear staining in blue) show co-localization of galectin-3 and endoglin (yellow color). Representative images of five different experiments are shown.

The interaction and association results between galectin-3 and endoglin shown above, prompted us to test whether galectin-3 co-localized with membrane-bound endoglin at the cellular level. Supporting this hypothesis, co-localization of endogenous galectin-3 and endoglin in the plasma membrane of HUVECs under basal conditions was observed by confocal microscopy (Figure 2).

Immunostaining of galectin-3 was detected at the membrane and intracellular compartments, whereas endoglin signal was mainly located at the cell surface (Figure 2A). Some of these cells showed a clear membrane co-localization of galectin-3 and endoglin, as depicted in the merged images (Figure 2B, yellow staining). Because both endoglin and galectin-3 are expressed by endothelial cells [28,52], their additional co-localization within the same secretory pathway (for example in the Golgi) cannot be excluded. This would be compatible with the occasional perinuclear yellow staining observed (Figure 2B). Control labeling in the absence of primary antibodies did not show any positive signals (Supplementary Materials Figure S1), ruling out a potential artifact of immunofluorescent secondary antibodies. Taken together, the above results demonstrate the interaction and association between galectin-3 and endoglin.

### 3.3. TRIM21 Interacts with Endoglin in Cells

**Figure 3:**
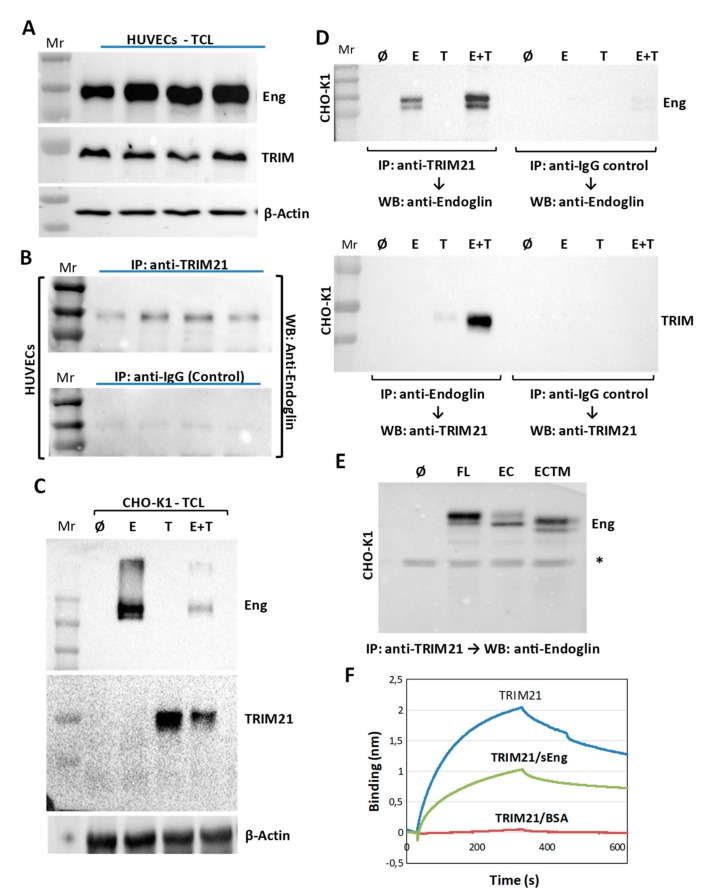
Protein–protein association between TRIM21 and endoglin. (**A**–**E**) Co-immunoprecipitation of TRIM21 and endoglin. A,B. HUVEC monolayers were lysed and total cell lysates (TCL) were subjected to SDS-PAGE under reducing (for TRIM21 detection) or nonreducing (for endoglin detection) conditions, followed by Western blot (WB) analysis using antibodies to endoglin, TRIM21 or β-actin (**A**). HUVECs lysates were subjected to immunoprecipitation (IP) with anti-TRIM21 or negative control antibodies, followed by WB analysis with anti-endoglin (**B**). C,D. CHO-K1 cells were transiently transfected with pDisplay–HA–Mock (Ø), pDisplay–HA–EngFL (**E**) or pcDNA3.1–HA–hTRIM21 (T) expression vectors, as indicated. Total cell lysates (TCL) were subjected to SDS-PAGE under nonreducing conditions and WB analysis using specific antibodies to endoglin, TRIM21, and β-actin (**C**). Cell lysates were subjected to immunoprecipitation (IP) with anti-TRIM21 or anti-endoglin antibodies, followed by SDS-PAGE under reducing (upper panel) or nonreducing (lower panel) conditions and WB analysis with anti-TRIM21 or anti-endoglin antibodies. Negative controls of appropriate IgG were included (**D**). E. CHO-K1 cells were transiently transfected with pcDNA3.1–HA–hTRIM21 and pDisplay–HA–Mock (Ø), pDisplay–HA–EngFL (FL; full-length), pDisplay–HA–EngEC (EC; cytoplasmic-less) or pDisplay–HA–EngTMEC (TMEC; cytoplasmic-less) expression vectors, as indicated. Cell lysates were subjected to immunoprecipitation with anti-TRIM21, followed by SDS-PAGE under reducing conditions and WB analysis with anti-endoglin antibodies, as indicated. The asterisk indicates the presence of a nonspecific band. Mr, molecular reference; Eng, endoglin; TRIM, TRIM21. (**F**) Protein–protein interactions between TRIM21 and endoglin using Bio-layer interferometry (BLItz). The Ni–NTA biosensors tips were loaded with 5.4 µM recombinant human TRIM21/6xHis at the N-terminus (R052), and protein binding was measured against 0.1% BSA in PBS (negative control) or 4.1 µM soluble endoglin (sEng). Kinetic sensorgrams were obtained using a single channel ForteBioBLItzTM instrument.

The TRIM family is a highly conserved group of E3 ubiquitin ligase proteins that have been implicated in the regulation of the TGF-β-dependent cellular, as well as in the ubiquitination and degradation of several components of TGF-β signaling pathway [55]. Unfortunately, little is known about the connection between the TRIM family and the TGF-β auxiliary receptor endoglin of endothelial cells. Among TRIM family members, TRIM21 acts not only as an E3 ubiquitin ligase on different target proteins, but also as a substrate in auto-ubiquitination, both leading to protein degradation [56,57,58]. In addition, TRIM21 is involved in cancer [59,60], inflammation [61,62,63,64], intracellular immunity, and autoimmunity [47,65,66,67]. Here, we validated the protein screen data for TRIM21, using two-way co-immunoprecipitation analysis of endoglin and TRIM21 in HUVECs and co-transfected CHO-K1 cells (Figure 3).

Endoglin and TRIM21 were readily detected in whole HUVECs lysates, as shown by WB analysis (Figure 3A). Co-immunoprecipitation studies of these lysates showed that endoglin was present in TRIM21 immunoprecipitates, but not in a negative control (Figure 3B). In our hands, TRIM21 could not be detected in endoglin immunoprecipitates from HUVECs (data not shown). For this reason, a proteomic approach was used. Thus, HUVEC lysates were subjected to immunoprecipitation with anti-endoglin antibodies, and the total proteins associated with immunoprecipitates were separated by SDS-PAGE, followed by trypsin digestion and proteomic analysis by mass spectrometry. As shown in Supplementary Materials Table S1, TRIM21 was found among the putative endoglin-interacting proteins identified by this technique. Further support for the endoglin/TRIM21 association was obtained from co-transfection experiments in CHO-K1 cells (Figure 3C,D). Figure 3C shows that endoglin and TRIM21 were clearly expressed upon transfection of CHO-K1 cells, as evidenced by WB analysis of total cell extracts. Co-immunoprecipitation studies of these cell lysates showed that endoglin was present in TRIM21 immunoprecipitates (Figure 3D, upper panel). Conversely, TRIM21 was detected in endoglin immunoprecipitates, as shown by WB analysis (Figure 3D, lower panel). In addition, co-immunoprecipitation studies of CHO-K1 cells, previously co-transfected with TRIM21 and two different truncated constructs (pDisplay–HA–EngEC and pDisplay–HA–EngTMEC), both devoid of the cytoplasmic tail of endoglin, revealed that these mutant proteins were present in TRIM21 immunoprecipitates, suggesting that the cytoplasmic domain of endoglin is not required for interaction with TRIM21 (Figure 3E).

Further evidence for the interaction between TRIM21 and endoglin was obtained by biolayer interferometry. The 6His-tagged TRIM21 was immobilized on Ni–NTA biosensor tips, and protein interactions were measured against soluble endoglin or its corresponding control buffer (Figure 3F). The association kinetics was monitored, revealing a specific interaction between TRIM21 and endoglin, as compared to the background signal of BSA.

**Figure 4:**
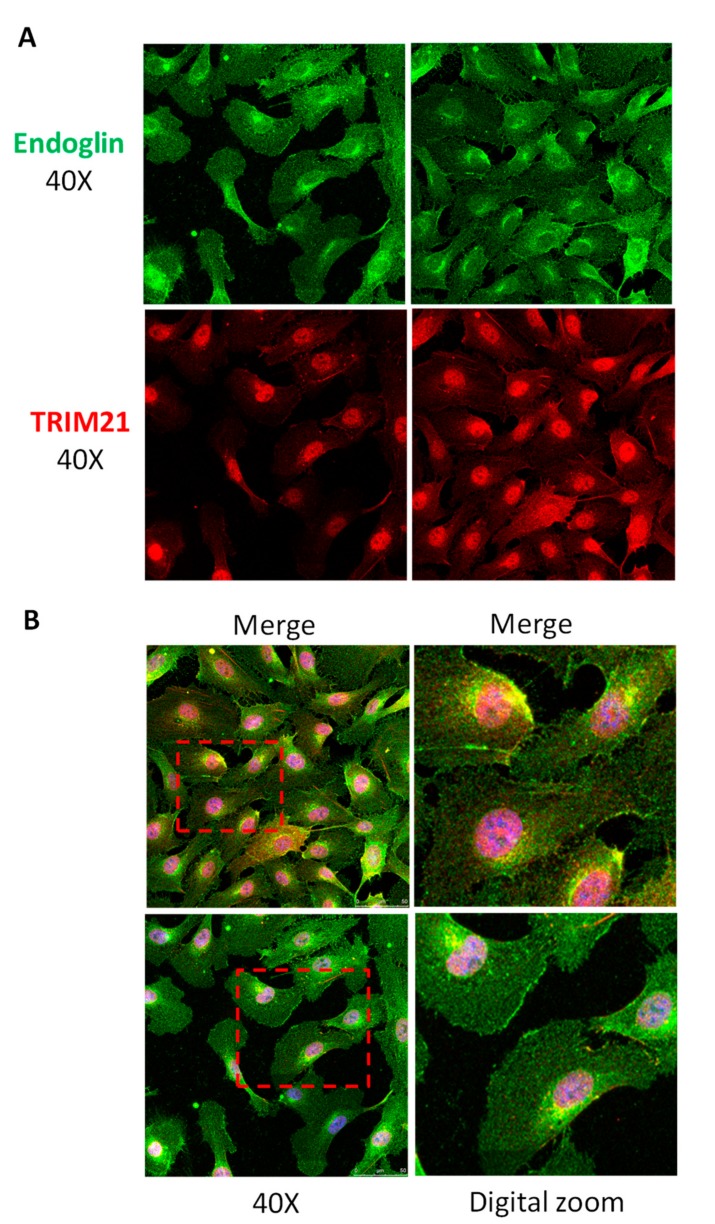
TRIM21 and endoglin co-localize in human endothelial cells. HUVEC monolayers were fixed with paraformaldehyde, permeabilized with Triton X-100, incubated with the mouse mAb P4A4 anti-endoglin, washed and incubated with a rabbit monoclonal anti-TRIM21 antibody (#92043). TRIM21 and endoglin were detected by immunofluorescence upon incubation with Alexa 647 goat anti-rabbit IgG (red staining) and Alexa 488 goat anti-mouse IgG (green staining) secondary antibodies, respectively. (**A**) Single staining of TRIM21 (red) and endoglin (green) at the indicated magnifications. (**B**) Merge images plus DAPI (nuclear staining in blue) show co-localization of TRIM21 and endoglin (yellow color). Representative images of four different experiments are shown.

Next, we tested whether TRIM21 co-localized with membrane-bound endoglin at the cellular level. As shown by confocal microscopy, immunostaining of TRIM21 was detected at the nucleus, membrane, and cytosolic compartments, whereas endoglin signal was mainly located at the cell surface (Figure 4). Endogenous TRIM21 and endoglin co-localized in the plasma membrane of HUVECs under basal conditions (Figure 4A). As depicted in the merged images (Figure 4B, yellow staining), some cells showed a clear membrane and perinuclear co-localization of TRIM21 and endoglin. Control labeling in the absence of primary antibodies did not show any positive signal (Figure S1), ruling out a potential artifact of immunofluorescent secondary antibodies.

Taken together, the above results demonstrate for the first time the interaction and association between TRIM21 and endoglin.

## 4. Discussion

The protein array screening allowed us to test a total of >9000 putative interactions with the extracellular region of endoglin using commercially available sEng. This is at variance with other screening studies which have previously used either the cytoplasmic region of endoglin or the full- length protein as a bait [9,68,69,70,71]. Here, we identified 22 interactors that passed our stringent criteria. They are located in different subcellular compartments, including plasma membrane, cytosol, cytoskeleton or extracellular secreted proteins. Surprisingly, some of the identified interactors are not membrane or secreted proteins, something that would be expected from the extracellular region of a type I membrane protein as endoglin. Several interactors are located in the nucleus, suggesting a yet-to-discover function for endoglin. Compatible with this hypothesis, endoglin deficiency or overexpression in human cells can regulate the expression of a wide range of target genes at the transcriptional level [72,73,74]. While the localization of endoglin in the nucleus has not been demonstrated, the intriguing functional impact of endoglin interactions with the newly identified nuclear partners remains to be explored.

Here we have confirmed the interaction between endoglin and two previously unrecognized partners, galectin-3 and TRIM21. Endoglin is a glycoprotein with several consensus N- and O- linked glycosylation sites [28]. Therefore, its capacity to bind galectin-3 fully agrees with the fact that this lectin contains a carbohydrate-recognition-binding domain involved in the interaction with different membrane glycoproteins [52]. Given the key role of endoglin in the vascular system, one of the reasons for choosing galectin-3 was its widely reported involvement in cardiovascular disease, including cardiac fibrosis and inflammation, pathological angiogenesis, aortic stenosis, abdominal aortic aneurysm, endothelial dysfunction, cardiovascular remodeling or hypertension [75,76,77,78,79,80]. Interestingly, both galectin-3 and endoglin can be expressed as a secreted biomarker in circulation and both have been involved in endothelial dysfunction and inflammation. Galectin-3 has been reported to exacerbate endothelial dysfunction via oxidized-LDL [79] and likely through oxidized-HDL-induced LOX-1 activation [81]. Further, sEng synergizes with hypercholesterolemia to aggravate endothelial and vessel wall dysfunction [26]. Interestingly, galectin-3 and sEng are increased in preeclampsia a systemic syndrome associated with inflammation, endothelial dysfunction and anti-angiogenic activity [18,43,82]. Additionally, galectin-3 and sEng regulate endothelial cell migration and angiogenic activity [22,83,84,85,86]. Because abnormal levels of galectin-3 and sEng are found in several endothelium-related pathological conditions, these results suggest that these soluble proteins may associate with each other, acting in concert to regulate endothelial function in vascular-related pathologies. In this regard, the galectin-3/endoglin interaction in the endothelial context is supported by a recent and preliminary work where endoglin was identified, among other glycoproteins, after a purification process of HUVEC extracts through a galectin-3 affinity column [86]. In addition, galectin-3 was detected in endoglin immunoprecipitates from lysates of HUVECs previously treated with recombinant galectin-3 [87]. Galectin-3 is a relatively abundant component of the tumor microenvironment, where it has been found to regulate critical aspects of cancer biology, such as metastasis, apoptosis, and immune surveillance [88]. Since elevated levels of sEng are also found in different types of cancer [9,89], it is tempting to speculate on the involvement of sEng/galectin-3 interaction in tumor progression and metastasis.

We have also shown that endoglin can interact with TRIM21, a protein involved in multiple cell functions. As other members of the TRIM family, TRIM21 can act as a ubiquitination enzyme targeting for degradation different proteins [56,57,58,90]. The TRIM family has been shown to ubiquitinate different components of the TGF-β signaling pathway [55,91], and our results suggest for the first time that TRIM21 may target for degradation the TGF-β auxiliary receptor endoglin. This interesting line of research should be explored in future experiments and may be key to understanding the described long half-life of this relatively stable glycoprotein [92]. Several reports have shown the involvement of TRIM21 in cancer development [58,59,60,93], a characteristic shared by other TRIM family members [55] and, interestingly, by endoglin [9,89]. TRIM21 functions as a tumor suppressor in breast cancer and high expression levels of TRIM21 are associated with longer overall survival of breast cancer patients [58,60]. Notably, a similar role for endoglin as a tumor suppressor in breast, prostate, esophageal, and skin carcinomas has been described [13,14,15,16]. These data suggest that, upon interacting with each other, endoglin and TRIM21 may act in concert to regulate cancer development. In line with this, both TRIM21 [58] and endoglin [94] appear to induce the epithelial–mesenchymal transition (EMT), a key process in cancer development. In addition, galectin-3 can also induce the mesenchymal transition of endothelial cells [95]. Another interesting finding of our studies is the presence of TRIM21 in endothelial cells, considering that the expression and function of TRIM21 in this cell lineage has been poorly addressed so far. Very recently, it has been reported that TRIM21 from human lung endothelial cells displays a modulatory role during the inflammatory response to lipopolysaccharide (LPS), suggesting that TRIM21 is a potential therapeutic target in sepsis-induced endothelial dysfunction [96,97]. Similarly, endothelial endoglin, which is markedly increased during inflammation [2,98], regulates several inflammation-related processes such as LPS-induced leukocyte extravasation [24] or cell-mediated vascular repair [99]. In addition, patients with septic shock present higher levels of circulating sEng compared to healthy individuals [100], while it has been reported that sEng contributes to endothelial dysfunction in animal models [26,27]. Further studies are needed to elucidate the exact role that the TRIM21/endoglin interaction plays in the complex role of endothelial cells during the inflammatory process.

## 5. Conclusions

In summary, here we identified more than twenty novel interactors of the extracellular region of endoglin, of which endoglin/galectin-3 and endoglin/TRIM21 associations were further characterized. Given the key involvement of membrane-bound and soluble endoglin in different pathophysiological contexts, these findings open up a new research avenue to better understand the mechanism of action of endoglin. Future independent studies remain to be performed to assess the functional significance of the endoglin interactions with these novel partners.

## Figures and Tables

**Table 1:** Human protein-array analysis of endoglin interactors^1^.

Accession #	Protein Name	Cellular Compartment
NM_172160.1	Potassium voltage-gated channel, shaker-related subfamily, beta member 1 (KCNAB1), transcript variant 1	Plasma membrane
Q14722		
NM_138565.1	Cortactin (CTTN), transcript variant 2	Plasma membrane
Q14247		
BC036123.1	Stromal membrane-associated protein 1 (SMAP1)	Plasma membrane
Q8IYB5		
NM_173822.1	Family with sequence similarity 126, member B (FAM126B)	Plasma membrane, cytosol
Q8IXS8		
BC047536.1	Sciellin (SCEL)	Plasma membrane, extracellular or secreted
O95171		
**BC068068.1**	**Galectin-3**	Plasma membrane, mitochondrion, nucleus, extracellular or secreted
**P17931**		
BC001247.1	Actin-binding LIM protein 1 (ABLIM1)	Cytoskeleton
O14639		
NM_198943.1	Family with sequence similarity 39, member B (FAM39B)	Endosome, cytoskeleton
Q6VEQ5		
NM_005898.4	Cell cycle associated protein 1 (CAPRIN1), transcript variant 1	Cytosol
Q14444		
BC002559.1	YTH domain family, member 2 (YTHDF2)	Nucleus, cytosol
Q9Y5A9		
**NM_003141.2**	**Tripartite motif-containing 21 (TRIM21)**	Nucleus, cytosol
**P19474**		
BC025279.1	Scaffold attachment factor B2 (SAFB2)	Nucleus
Q14151		
BC031650.1	Putative E3 ubiquitin-protein ligase SH3RF2	Nucleus
Q8TEC5		
BC034488.2	ATP-binding cassette, sub-family F (GCN20), member 1 (ABCF1)	Nucleus
Q8NE71		
BC040946.1	Spliceosome-associated protein CWC15 homolog (HSPC148)	Nucleus
Q9P013		
NM_003609.2	HIRA interacting protein 3 (HIRIP3)	Nucleus
Q9BW71		
NM_005572.1	Lamin A/C (LMNA), transcript variant 2	Nucleus
P02545		
NM_006479.2	RAD51 associated protein 1 (RAD51AP1)	Nucleus
Q96B01		
NM_014321.2	Origin recognition complex, subunit 6 like (yeast) (ORC6L)	Nucleus
Q9Y5N6		
NM_015138.2	RNA polymerase-associated protein RTF1 homolog (RTF1)	Nucleus
Q92541		
NM_032141.1	Coiled-coil domain containing 55 (CCDC55), transcript variant 1	Nucleus
Q9H0G5		
BC012289.1	Protein PRRC2B, KIAA0515	Data not available
Q5JSZ5		

^1^ Microarrays containing over 9000 unique human proteins were screened using recombinant sEng as a probe. Protein interactors showing the highest scores (Z-score ≥2.0) are listed. GeneBank (https://www.ncbi.nlm.nih.gov/genbank/) and UniProtKB (https://www.uniprot.org/help/uniprotkb) accession numbers are indicated with a yellow or green background, respectively. The cellular compartment of each protein was obtained from the UniProtKB webpage. Proteins selected for further studies (TRIM21 and galectin-3) are indicated in bold type with blue background.

## Supplementary Materials

The following is available online at https://www.mdpi.com/2073-4409/8/9/1082/s1, Figure S1: Immunofluorescence staining of galectin-3, TRIM21, and endoglin in human endothelial cells, Table S1: Proteomic analysis of endoglin-associated proteins.
