# Supplementary material for: Endoglin Protein Interactome Profiling Identifies TRIM21 and Galectin-3 as New Binding Partners
Source: Cells. 2019 Sep 13;8(9):1082. doi: 10.3390/cells8091082 (PMC6769930; doi:10.3390/cells8091082)
Supplement: Supplementary file 1 [file cells-08-01082-s001.pdf]

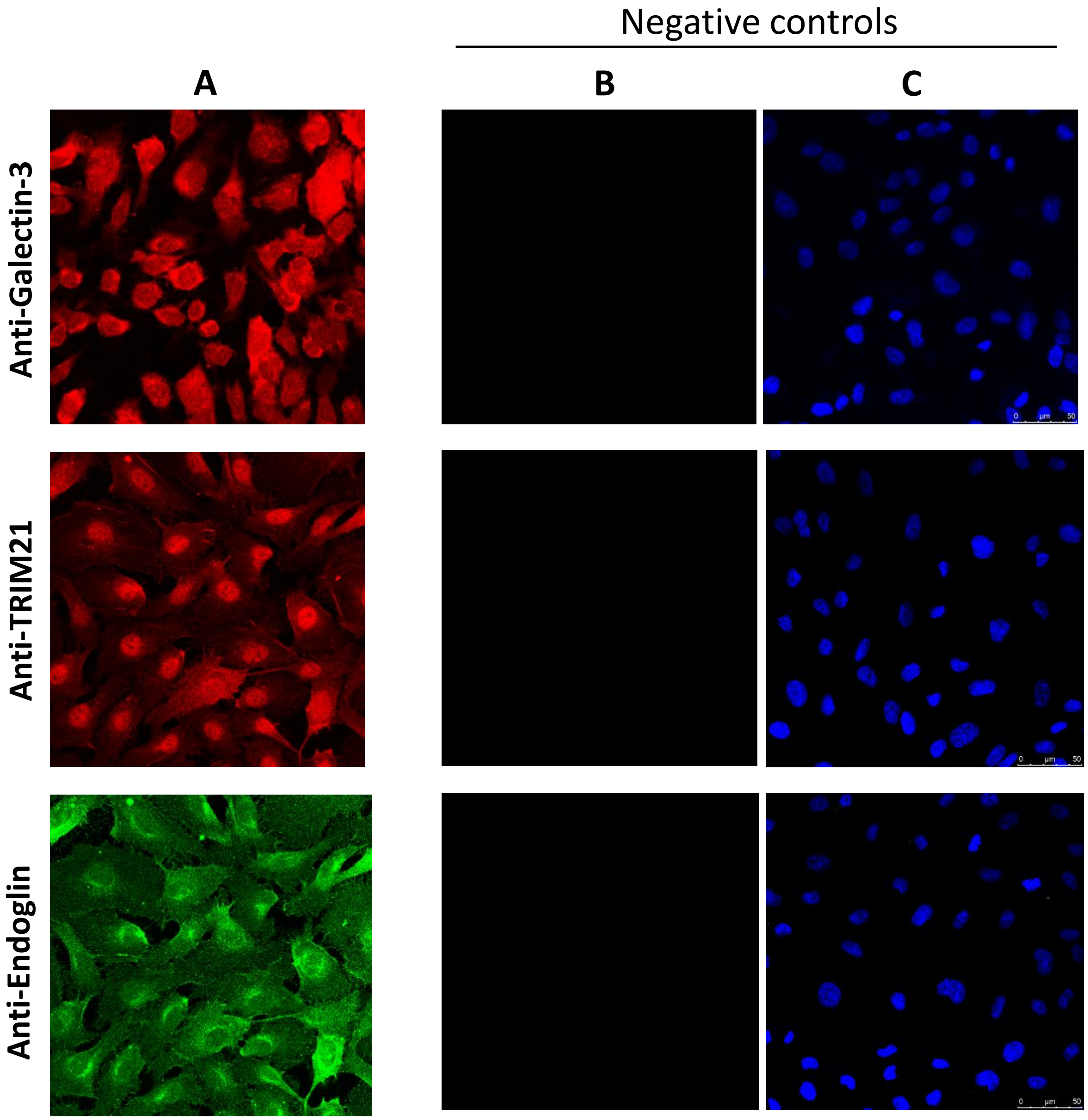

Supplementary Figure 1. Immunofluorescence staining of galectin-3, TRIM21 and endoglin in human endothelial cells. HUVEC monolayers were fixed, permeabilized, and incubated with a rabbit polyclonal anti-galectin-3 antibody, a rabbit monoclonal anti-TRIM21 antibody or a mouse monoclonal anti-endoglin antibody as in Figures 2 and 4. **A.** Galectin-3 and TRIM21 were detected by immunofluorescence upon incubation with Alexa 647 goat anti-rabbit IgG (red staining), whereas endoglin was visualized upon incubation with Alexa 488 goat anti-mouse IgG (green staining). **B,C.** The corresponding negative controls in the absence of primary antibody, without (**B**) or with (**C**) DAPI staining (blue) are shown. Representative images of five different experiments are shown.

Supplementary Table S1. Proteomic analysis of endoglin-associated proteins*

Accession	Description	ΣCoverage	Σ# Proteins	Σ# Unique Peptides	Σ# Peptides	Σ# PSMs
P06493	Cyclin-dependent kinase 1 OS=Homo sapiens GN=CDK1 PE=1 SV=3 - [CDK1_HUMAN]	26.60	4	6	6	25
Q96HC4	PDZ and LIM domain protein 5 OS=Homo sapiens GN=PDLIM5 PE=1 SV=5 - [PDLI5_HUMAN]	20.81	1	2	11	54
P05141	ADP/ATP translocase 2 OS=Homo sapiens GN=SLC25A5 PE=1 SV=7 - [ADT2_HUMAN]	19.46	3	2	6	15
Q92945	Far upstream element-binding protein 2 OS=Homo sapiens GN=KHSRP PE=1 SV=4 - [FUBP2_HUMAN]	17.72	1	2	12	51
Q9NXV6	CDKN2A-interacting protein OS=Homo sapiens GN=CDKN2AIP PE=1 SV=3 - [CARF_HUMAN]	15.17	1	2	6	11
Q05682	Caldesmon OS=Homo sapiens GN=CALD1 PE=1 SV=3 - [CALD1_HUMAN]	15.01	1	2	15	67
O75396	Vesicle-trafficking protein SEC22b OS=Homo sapiens GN=SEC22B PE=1 SV=4 - [SC22B_HUMAN]	14.88	1	2	3	17
Q9Y3D9	28S ribosomal protein S23, mitochondrial OS=Homo sapiens GN=MRPS23 PE=1 SV=2 - [RT23_HUMAN]	14.74	1	3	3	7
Q15005	Signal peptidase complex subunit 2 OS=Homo sapiens GN=SPCS2 PE=1 SV=3 - [SPCS2_HUMAN]	13.72	1	2	2	7
Q9UDY2	Tight junction protein ZO-2 OS=Homo sapiens GN=TJP2 PE=1 SV=2 - [ZO2_HUMAN]	11.85	1	3	14	33
O15231	Zinc finger protein 185 OS=Homo sapiens GN=ZNF185 PE=1 SV=3 - [ZN185_HUMAN]	11.61	1	3	6	6
Q99988	Growth/differentiation factor 15 OS=Homo sapiens GN=GDF15 PE=2 SV=3 - [GDF15_HUMAN]	11.36	2	3	3	5
Q7L5D6	UPF0363 protein C7orf20 OS=Homo sapiens GN=C7orf20 PE=1 SV=1 - [CG020_HUMAN]	11.01	1	3	3	7
A0A075B6P5	Immunoglobulin kappa variable 2-28 OS=Homo sapiens GN=IGKV2-28 PE=3 SV=1 - [KV228_HUMAN]	10.83	4	1	1	6
Q96FQ6	Protein S100-A16 OS=Homo sapiens GN=S100A16 PE=1 SV=1 - [S10AG_HUMAN]	10.68	1	1	1	2
Q9P2A4	ABI gene family member 3 OS=Homo sapiens GN=ABI3 PE=1 SV=2 - [ABI3_HUMAN]	10.66	1	3	3	7
P05114	Non-histone chromosomal protein HMG-14 OS=Homo sapiens GN=HMGN1 PE=1 SV=3 - [HMGN1_HUMAN]	10.00	1	1	1	3
P17655	Calpain-2 catalytic subunit OS=Homo sapiens GN=CAPN2 PE=1 SV=6 - [CAN2_HUMAN]	10.00	1	2	6	12
P14317	Hematopoietic lineage cell-specific protein OS=Homo sapiens GN=HCLS1 PE=1 SV=3 - [HCLS1_HUMAN]	9.67	1	1	4	4
Q9NR31	GTP-binding protein SAR1a OS=Homo sapiens GN=SAR1A PE=1 SV=1 - [SAR1A_HUMAN]	9.60	2	2	2	3
P0CG05	Ig lambda-2 chain C regions OS=Homo sapiens GN=IGLC2 PE=1 SV=1 - [LAC2_HUMAN]	9.43	2	1	1	1
Q03252	Lamin-B2 OS=Homo sapiens GN=LMNB2 PE=1 SV=4 - [LMNB2_HUMAN]	9.35	1	1	6	11
A1KXE4	Protein FAM168B OS=Homo sapiens GN=FAM168B PE=1 SV=1 - [F168B_HUMAN]	9.23	1	1	1	4
Q9UL46	Proteasome activator complex subunit 2 OS=Homo sapiens GN=PSME2 PE=1 SV=4 - [PSME2_HUMAN]	9.21	1	2	2	7
Q96EM0	Trans-3-hydroxy-L-proline dehydratase OS=Homo sapiens GN=L3HYPDH PE=1 SV=2 - [T3HPD_HUMAN]	8.76	1	2	2	8
P10599	Thioredoxin OS=Homo sapiens GN=TXN PE=1 SV=3 - [THIO_HUMAN]	8.57	1	1	1	1
Q8WXX5	DnaJ homolog subfamily C member 9 OS=Homo sapiens GN=DNAJC9 PE=1 SV=1 - [DNJC9_HUMAN]	8.46	1	2	2	7
Q9NSD9	Phenylalanine--tRNA ligase beta subunit OS=Homo sapiens GN=FARSB PE=1 SV=3 - [SYFB_HUMAN]	8.32	1	2	5	7
Q13435	Splicing factor 3B subunit 2 OS=Homo sapiens GN=SF3B2 PE=1 SV=2 - [SF3B2_HUMAN]	7.82	1	3	7	11
O76003	Glutaredoxin-3 OS=Homo sapiens GN=GLRX3 PE=1 SV=2 - [GLRX3_HUMAN]	7.76	1	1	1	1
Q92600	Cell differentiation protein RCD1 homolog OS=Homo sapiens GN=RQCD1 PE=1 SV=1 - [RCD1_HUMAN]	7.69	1	2	2	2
Q6P1K2	Polyamine-modulated factor 1 OS=Homo sapiens GN=PMF1 PE=1 SV=2 - [PMF1_HUMAN]	7.32	1	1	1	3
P60981	Destrin OS=Homo sapiens GN=DSTN PE=1 SV=3 - [DEST_HUMAN]	7.27	1	1	1	1
Q5TBB1	Ribonuclease H2 subunit B OS=Homo sapiens GN=RNASEH2B PE=1 SV=1 - [RNH2B_HUMAN]	7.05	1	2	2	2
Q8NDC0	MAPK-interacting and spindle-stabilizing protein-like OS=Homo sapiens GN=MAPK1IP1L PE=1 SV=4 - [MISSL_HUMAN]	6,94	1	1	1	12
Q9NX58	Cell growth-regulating nucleolar protein OS=Homo sapiens GN=LYAR PE=1 SV=2 - [LYAR_HUMAN]	6.86	1	2	2	2
Q9UL25	Ras-related protein Rab-21 OS=Homo sapiens GN=RAB21 PE=1 SV=3 - [RAB21_HUMAN]	6.67	1	1	1	2
Q9Y676	28S ribosomal protein S18b, mitochondrial OS=Homo sapiens GN=MRPS18B PE=1 SV=1 - [RT18B_HUMAN]	6.59	1	1	1	1
O43583	Density-regulated protein OS=Homo sapiens GN=DENR PE=1 SV=2 - [DENR_HUMAN]	6.57	1	1	1	2
A6NDG6	Phosphoglycolate phosphatase OS=Homo sapiens GN=PGP PE=1 SV=1 - [PGP_HUMAN]	6.54	1	2	2	5
Q9Y3A3	Mps one binder kinase activator-like 3 OS=Homo sapiens GN=MOBKL3 PE=1 SV=1 - [MOBL3_HUMAN]	6.22	1	1	1	1
P61626	Lysozyme C OS=Homo sapiens GN=LYZ PE=1 SV=1 - [LYSC_HUMAN]	6.08	1	1	1	3
Q8WUD4	Coiled-coil domain-containing protein 12 OS=Homo sapiens GN=CCDC12 PE=1 SV=1 - [CCD12_HUMAN]	6.02	1	1	1	3
Q15691	Microtubule-associated protein RP/EB family member 1 OS=Homo sapiens GN=MAPRE1	5.97	1	1	1	7

	PE=1 SV=3 - [MARE1_HUMAN]					
Q9Y6G5	COMM domain-containing protein 10 OS=Homo sapiens GN=COMMD10 PE=1 SV=1 - [COMDA_HUMAN]	5.94	1	1	1	1
O95299	NADH dehydrogenase [ubiquinone] 1 alpha subcomplex subunit 10, mitochondrial OS=Homo sapiens GN=NDUFA10 PE=1 SV=1 - [NDUAA_HUMAN]	5.92	1	2	2	6
P49023	Paxillin OS=Homo sapiens GN=PXN PE=1 SV=3 - [PAXI_HUMAN]	5.92	2	2	2	2
Q86X55	Histone-arginine methyltransferase CARM1 OS=Homo sapiens GN=CARM1 PE=1 SV=3 - [CARM1_HUMAN]	5.76	1	1	1	1
Q8NEJ9	Neuroguidin OS=Homo sapiens GN=NGDN PE=1 SV=1 - [NGDN_HUMAN]	5.71	1	1	1	1
Q00403	Transcription initiation factor IIB OS=Homo sapiens GN=GTF2B PE=1 SV=1 - [TF2B_HUMAN]	5.70	1	2	2	5
O00161	Synaptosomal-associated protein 23 OS=Homo sapiens GN=SNAP23 PE=1 SV=1 - [SNP23_HUMAN]	5.69	1	1	1	1
Q00796	Sorbitol dehydrogenase OS=Homo sapiens GN=SORD PE=1 SV=4 - [DHSO_HUMAN]	5.60	1	1	1	1
P18085	ADP-ribosylation factor 4 OS=Homo sapiens GN=ARF4 PE=1 SV=3 - [ARF4_HUMAN]	5.56	1	1	1	1
Q5RKV6	Exosome complex exonuclease MTR3 OS=Homo sapiens GN=EXOSC6 PE=1 SV=1 - [EXOS6_HUMAN]	5.51	1	1	1	1
Q9NQT5	Exosome complex exonuclease RRP40 OS=Homo sapiens GN=EXOSC3 PE=1 SV=3 - [EXOS3_HUMAN]	5.45	1	1	1	11
Q9UKX7	Nuclear pore complex protein Nup50 OS=Homo sapiens GN=NUP50 PE=1 SV=2 - [NUP50_HUMAN]	5.34	1	2	2	3
Q9NRG1	Phosphoribosyltransferase domain-containing protein 1 OS=Homo sapiens GN=PRTFDC1 PE=1 SV=1 - [PRDC1_HUMAN]	5,33	1	1	1	3
O14530	Thioredoxin domain-containing protein 9 OS=Homo sapiens GN=TXNDC9 PE=1 SV=2 - [TXND9_HUMAN]	5.31	1	1	1	1
Q9Y5Y2	Cytosolic Fe-S cluster assembly factor NUBP2 OS=Homo sapiens GN=NUBP2 PE=1 SV=1 - [NUBP2_HUMAN]	5.17	1	1	1	1
P07305	Histone H1.0 OS=Homo sapiens GN=H1F0 PE=1 SV=3 - [H10_HUMAN]	5.15	1	1	1	1
Q8NBT2	Kinetochore protein Spc24 OS=Homo sapiens GN=SPC24 PE=1 SV=2 - [SPC24_HUMAN]	5.08	2	1	1	3
Q15434	RNA-binding motif, single-stranded-interacting protein 2 OS=Homo sapiens GN=RBMS2 PE=1 SV=1 - [RBMS2_HUMAN]	4.91	3	2	2	2
Q6I9Y2	THO complex subunit 7 homolog OS=Homo sapiens GN=THOC7 PE=1 SV=3 - [THOC7_HUMAN]	4.90	1	1	1	3
O14602	Eukaryotic translation initiation factor 1A, Y-chromosomal OS=Homo sapiens GN=EIF1AY PE=1 SV=4 - [IF1AY_HUMAN]	4.86	2	1	1	2
P57088	Transmembrane protein 33 OS=Homo sapiens GN=TMEM33 PE=1 SV=2 - [TMM33_HUMAN]	4.86	1	1	1	1
Q7Z7C8	Transcription initiation factor TFIID subunit 8 OS=Homo sapiens GN=TAF8 PE=1 SV=1 - [TAF8_HUMAN]	4.84	1	1	1	2
Q9H939	Proline-serine-threonine phosphatase-interacting protein 2 OS=Homo sapiens GN=PSTPIP2 PE=1 SV=4 - [PPIP2_HUMAN]	4.79	1	2	2	2
O43402	Neighbor of COX4 OS=Homo sapiens GN=COX4NB PE=1 SV=1 - [CX4NB_HUMAN]	4,76	1	1	1	1
Q8TBC4	NEDD8-activating enzyme E1 catalytic subunit OS=Homo sapiens GN=UBA3 PE=1 SV=2 - [UBA3_HUMAN]	4.75	1	1	1	1
O95999	B-cell lymphoma/leukemia 10 OS=Homo sapiens GN=BCL10 PE=1 SV=1 - [BCL10_HUMAN]	4.72	1	1	1	4
Q07002	Cell division protein kinase 18 OS=Homo sapiens GN=CDK18 PE=1 SV=3 - [CDK18_HUMAN]	4.66	3	2	2	3
Q9Y6A4	UPF0468 protein C16orf80 OS=Homo sapiens GN=C16orf80 PE=1 SV=1 - [CP080_HUMAN]	4.66	1	1	1	2
Q16270	Insulin-like growth factor-binding protein 7 OS=Homo sapiens GN=IGFBP7 PE=1 SV=1 - [IBP7_HUMAN]	4.61	1	1	1	1
P48060	Glioma pathogenesis-related protein 1 OS=Homo sapiens GN=GLIPR1 PE=1 SV=3 - [GLIP1_HUMAN]	4.51	1	1	1	5
Q53GQ0	Estradiol 17-beta-dehydrogenase 12 OS=Homo sapiens GN=HSD17B12 PE=1 SV=2 - [DHB12_HUMAN]	4.49	1	1	1	1
Q96MF7	E3 SUMO-protein ligase NSE2 OS=Homo sapiens GN=NSMCE2 PE=1 SV=2 - [NSE2_HUMAN]	4.45	1	1	1	2
Q9BXK1	Krueppel-like factor 16 OS=Homo sapiens GN=KLF16 PE=1 SV=1 - [KLF16_HUMAN]	4.37	1	1	1	1
Q9UBP6	tRNA (guanine-N(7)-)-methyltransferase OS=Homo sapiens GN=METTL1 PE=1 SV=1 - [TRMB_HUMAN]	4.35	1	1	1	9
Q9HD15	Steroid receptor RNA activator 1 OS=Homo sapiens GN=SRA1 PE=1 SV=1 - [SRA1_HUMAN]	4.24	1	1	1	1
Q5VWZ2	Lysophospholipase-like protein 1 OS=Homo sapiens GN=LYPLAL1 PE=1 SV=3 - [LYPL1_HUMAN]	4.22	1	1	1	2
O95865	N(G),N(G)-dimethylarginine dimethylaminohydrolase 2 OS=Homo sapiens GN=DDAH2 PE=1 SV=1 - [DDAH2_HUMAN]	4,21	1	1	1	1
Q14558	Phosphoribosyl pyrophosphate synthase-associated protein 1 OS=Homo sapiens GN=PRPSAP1 PE=1 SV=2 - [KPRA_HUMAN]	4.21	1	1	1	2
P61964	WD repeat-containing protein 5 OS=Homo sapiens GN=WDR5 PE=1 SV=1 - [WDR5_HUMAN]	4.19	1	1	1	2
P30281	G1/S-specific cyclin-D3 OS=Homo sapiens GN=CCND3 PE=1 SV=2 - [CCND3_HUMAN]	4.11	1	1	1	1
P09417	Dihydropteridine reductase OS=Homo sapiens GN=QDPR PE=1 SV=2 - [DHPR_HUMAN]	4.10	1	1	1	1
Q99426	Tubulin-folding cofactor B OS=Homo sapiens GN=TBCB PE=1 SV=2 - [TBCB_HUMAN]	4.10	1	1	1	2
Q9UBI1	COMM domain-containing protein 3 OS=Homo sapiens GN=COMMD3 PE=1 SV=1 - [COMD3_HUMAN]	4.10	1	1	1	1
Q9HD42	Charged multivesicular body protein 1a OS=Homo sapiens GN=CHMP1A PE=1 SV=1 -	4.08	1	1	1	4

	[CHM1A_HUMAN]					
O43324	Eukaryotic translation elongation factor 1 epsilon-1 OS=Homo sapiens GN=EEF1E1 PE=1 SV=1 - [MCA3_HUMAN]	4.02	1	1	1	3
Q7Z7H5	Transmembrane emp24 domain-containing protein 4 OS=Homo sapiens GN=TMED4 PE=1 SV=1 - [TMED4_HUMAN]	3.96	1	1	1	1
P15259	Phosphoglycerate mutase 2 OS=Homo sapiens GN=PGAM2 PE=1 SV=3 - [PGAM2_HUMAN]	3.95	2	1	1	5
Q15631	Translin OS=Homo sapiens GN=TSN PE=1 SV=1 - [TSN_HUMAN]	3.95	1	1	1	4
Q15041	ADP-ribosylation factor-like protein 6-interacting protein 1 OS=Homo sapiens GN=ARL6IP1 PE=1 SV=2 - [AR6P1_HUMAN]	3.94	1	1	1	2
Q9BYN8	28S ribosomal protein S26, mitochondrial OS=Homo sapiens GN=MRPS26 PE=1 SV=1 - [RT26_HUMAN]	3.90	1	1	1	1
P53801	Pituitary tumor-transforming gene 1 protein-interacting protein OS=Homo sapiens GN=PTTG1IP PE=1 SV=1 - [PTTG_HUMAN]	3.89	1	1	1	1
Q969G5	Protein kinase C delta-binding protein OS=Homo sapiens GN=PRKCDBP PE=1 SV=3 - [PRDBP_HUMAN]	3.83	1	1	1	1
Q15014	Mortality factor 4-like protein 2 OS=Homo sapiens GN=MORF4L2 PE=1 SV=1 - [MO4L2_HUMAN]	3.82	1	1	1	1
Q9P0W2	SWI/SNF-related matrix-associated actin-dependent regulator of chromatin subfamily E member 1-related OS=Homo sapiens GN=HMG20B PE=1 SV=1 - [HM20B_HUMAN]	3.79	1	1	1	1
Q9UJ70	N-acetyl-D-glucosamine kinase OS=Homo sapiens GN=NAGK PE=1 SV=4 - [NAGK_HUMAN]	3.78	1	1	1	1
Q96C19	EF-hand domain-containing protein D2 OS=Homo sapiens GN=EFHD2 PE=1 SV=1 - [EFHD2_HUMAN]	3.75	1	1	1	1
B9A064	Immunoglobulin lambda-like polypeptide 5 OS=Homo sapiens GN=IGLL5 PE=2 SV=2 - [IGLL5_HUMAN]	3.74	1	1	1	1
Q9H0J9	Poly [ADP-ribose] polymerase 12 OS=Homo sapiens GN=PARP12 PE=1 SV=1 - [PAR12_HUMAN]	3.71	1	1	1	1
P46821	Microtubule-associated protein 1B OS=Homo sapiens GN=MAP1B PE=1 SV=2 - [MAP1B_HUMAN]	3.69	3	2	7	22
Q9UHF1	Epidermal growth factor-like protein 7 OS=Homo sapiens GN=EGFL7 PE=1 SV=3 - [EGFL7_HUMAN]	3.66	1	1	1	1
Q9NVM6	DnaJ homolog subfamily C member 17 OS=Homo sapiens GN=DNAJC17 PE=1 SV=1 - [DJC17_HUMAN]	3.62	1	1	1	1
Q9H8W4	Pleckstrin homology domain-containing family F member 2 OS=Homo sapiens GN=PLEKHF2 PE=1 SV=1 - [PKHF2_HUMAN]	3.61	1	1	1	1
Q96AP7	Endothelial cell-selective adhesion molecule OS=Homo sapiens GN=ESAM PE=1 SV=1 - [ESAM_HUMAN]	3.59	1	1	1	1
O43148	mRNA cap guanine-N7 methyltransferase OS=Homo sapiens GN=RNMT PE=1 SV=1 - [MCES_HUMAN]	3.57	1	2	2	3
Q4G0F5	Vacuolar protein sorting-associated protein 26B OS=Homo sapiens GN=VPS26B PE=1 SV=2 - [VP26B_HUMAN]	3.57	1	1	1	1
O95766	UPF0550 protein C7orf28 OS=Homo sapiens GN=C7orf28A PE=1 SV=1 - [CG028_HUMAN]	3.53	1	1	1	1
Q9NX63	Coiled-coil-helix-coiled-coil-helix domain-containing protein 3, mitochondrial OS=Homo sapiens GN=CHCHD3 PE=1 SV=1 - [CHCH3_HUMAN]	3.52	1	1	1	1
P48426	Phosphatidylinositol-5-phosphate 4-kinase type-2 alpha OS=Homo sapiens GN=PIP4K2A PE=1 SV=2 - [PI42A_HUMAN]	3.45	1	1	1	2
Q15024	Exosome complex component RRP42 OS=Homo sapiens GN=EXOSC7 PE=1 SV=3 - [EXOS7_HUMAN]	3.44	1	1	1	1
Q9UKV3	Apoptotic chromatin condensation inducer in the nucleus OS=Homo sapiens GN=ACIN1 PE=1 SV=2 - [ACINU_HUMAN]	3.43	1	3	4	4
Q16594	Transcription initiation factor TFIID subunit 9 OS=Homo sapiens GN=TAF9 PE=1 SV=1 - [TAF9_HUMAN]	3.41	1	1	1	1
Q9P015	39S ribosomal protein L15, mitochondrial OS=Homo sapiens GN=MRPL15 PE=1 SV=1 - [RM15_HUMAN]	3.38	1	1	1	1
Q9Y580	RNA-binding protein 7 OS=Homo sapiens GN=RBM7 PE=1 SV=1 - [RBM7_HUMAN]	3.38	1	1	1	4
P48730	Casein kinase I isoform delta OS=Homo sapiens GN=CSNK1D PE=1 SV=2 - [KC1D_HUMAN]	3.37	1	1	1	2
Q96IU4	Abhydrolase domain-containing protein 14B OS=Homo sapiens GN=ABHD14B PE=1 SV=1 - [ABHEB_HUMAN]	3.33	1	1	1	1
Q96A35	39S ribosomal protein L24, mitochondrial OS=Homo sapiens GN=MRPL24 PE=1 SV=1 - [RM24_HUMAN]	3.24	1	1	1	2
Q96CS2	HAUS augmin-like complex subunit 1 OS=Homo sapiens GN=HAUS1 PE=1 SV=1 - [HAUS1_HUMAN]	3.24	1	1	1	3
P19404	NADH dehydrogenase [ubiquinone] flavoprotein 2, mitochondrial OS=Homo sapiens GN=NDUFV2 PE=1 SV=2 - [NDUV2_HUMAN]	3.21	1	1	1	1
P78549	Endonuclease III-like protein 1 OS=Homo sapiens GN=NTHL1 PE=1 SV=2 - [NTHL1_HUMAN]	3.21	1	1	1	2
Q9NQY0	Bridging integrator 3 OS=Homo sapiens GN=BIN3 PE=2 SV=1 - [BIN3_HUMAN]	3.16	1	1	1	1
P24539	ATP synthase subunit b, mitochondrial OS=Homo sapiens GN=ATP5F1 PE=1 SV=2 - [AT5F1_HUMAN]	3.13	1	1	1	3
Q6PK04	Coiled-coil domain-containing protein 137 OS=Homo sapiens GN=CCDC137 PE=1 SV=1 - [CC137_HUMAN]	3.11	1	1	1	1
Q96S97	Myeloid-associated differentiation marker OS=Homo sapiens GN=MYADM PE=1 SV=2 - [MYADM_HUMAN]	3.11	1	1	1	2
Q8WWC4	Uncharacterized protein C2orf47, mitochondrial OS=Homo sapiens GN=C2orf47 PE=1 SV=1 - [CB047_HUMAN]	3.09	1	1	1	2
Q8WTP8	Apoptosis-enhancing nuclease OS=Homo sapiens GN=AEN PE=1 SV=2 - [AEN_HUMAN]	3.08	1	1	1	1
P82650	28S ribosomal protein S22, mitochondrial OS=Homo sapiens GN=MRPS22 PE=1 SV=1 - [RT22_HUMAN]	3.06	1	1	1	3

Q92989	Polyribonucleotide 5'-hydroxyl-kinase Clp1 OS=Homo sapiens GN=CLP1 PE=1 SV=1 - [CLP1_HUMAN]	3.06	1	1	1	2
Q9Y4P1	Cysteine protease ATG4B OS=Homo sapiens GN=ATG4B PE=1 SV=2 - [ATG4B_HUMAN]	3.05	1	1	1	1
Q14914	Prostaglandin reductase 1 OS=Homo sapiens GN=PTGR1 PE=1 SV=2 - [PTGR1_HUMAN]	3.04	1	1	1	2
P23381	Tryptophanyl-tRNA synthetase, cytoplasmic OS=Homo sapiens GN=WARS PE=1 SV=2 - [SYWC_HUMAN]	2.97	1	1	1	2
P19474	52 kDa Ro protein OS=Homo sapiens GN=TRIM21 PE=1 SV=1 - [RO52_HUMAN]	2.95	1	1	1	1
P43034	Platelet-activating factor acetylhydrolase IB subunit alpha OS=Homo sapiens GN=PAFAH1B1 PE=1 SV=2 - [LIS1_HUMAN]	2.93	1	1	1	1
Q53H96	Pyrroline-5-carboxylate reductase 3 OS=Homo sapiens GN=PYCRL PE=1 SV=3 - [P5CR3_HUMAN]	2.92	1	1	1	1
O15160	DNA-directed RNA polymerases I and III subunit RPAC1 OS=Homo sapiens GN=POLR1C PE=1 SV=1 - [RPAC1_HUMAN]	2.89	1	1	1	2
P26440	Isovaleryl-CoA dehydrogenase, mitochondrial OS=Homo sapiens GN=IVD PE=1 SV=1 - [IVD_HUMAN]	2.84	1	1	1	1
Q9UBG3	Cornulin OS=Homo sapiens GN=CRNN PE=1 SV=1 - [CRNN_HUMAN]	2.83	1	1	1	1
Q7L4I2	Arginine/serine-rich coiled-coil protein 2 OS=Homo sapiens GN=RSRC2 PE=1 SV=1 - [RSRC2_HUMAN]	2.76	1	1	1	1
Q9BV68	RING finger protein 126 OS=Homo sapiens GN=RNF126 PE=1 SV=1 - [RN126_HUMAN]	2.76	1	1	1	1
O95983	Methyl-CpG-binding domain protein 3 OS=Homo sapiens GN=MBD3 PE=1 SV=1 - [MBD3_HUMAN]	2.75	1	1	1	1
Q9NXW2	DnaJ homolog subfamily B member 12 OS=Homo sapiens GN=DNAJB12 PE=1 SV=4 - [DJB12_HUMAN]	2.67	1	1	1	1
O94888	UBX domain-containing protein 7 OS=Homo sapiens GN=UBXN7 PE=1 SV=2 - [UBXN7_HUMAN]	2.66	1	1	1	2
Q9H3P2	Negative elongation factor A OS=Homo sapiens GN=WHSC2 PE=1 SV=3 - [NELFA_HUMAN]	2.65	1	1	1	3
Q9BRJ2	39S ribosomal protein L45, mitochondrial OS=Homo sapiens GN=MRPL45 PE=1 SV=2 - [RM45_HUMAN]	2.61	1	1	1	2
O43294	Transforming growth factor beta-1-induced transcript 1 protein OS=Homo sapiens GN=TGFB1I1 PE=1 SV=2 - [TGFI1_HUMAN]	2.60	1	1	1	1
Q9HA64	Ketosamine-3-kinase OS=Homo sapiens GN=FN3KRP PE=1 SV=2 - [KT3K_HUMAN]	2.59	1	1	1	3
Q15797	Mothers against decapentaplegic homolog 1 OS=Homo sapiens GN=SMAD1 PE=1 SV=1 - [SMAD1_HUMAN]	2.58	1	1	1	1
Q9BX67	Junctional adhesion molecule C OS=Homo sapiens GN=JAM3 PE=1 SV=1 - [JAM3_HUMAN]	2.58	1	1	1	2
P49247	Ribose-5-phosphate isomerase OS=Homo sapiens GN=RPIA PE=1 SV=3 - [RPIA_HUMAN]	2.57	1	1	1	2
Q13888	General transcription factor IIH subunit 2 OS=Homo sapiens GN=GTF2H2 PE=1 SV=1 - [TF2H2_HUMAN]	2.53	2	1	1	1
P50336	Protoporphyrinogen oxidase OS=Homo sapiens GN=PPOX PE=1 SV=1 - [PPOX_HUMAN]	2.52	1	1	1	2
Q96GM5	SWI/SNF-related matrix-associated actin-dependent regulator of chromatin subfamily D member 1 OS=Homo sapiens GN=SMARCD1 PE=1 SV=2 - [SMRD1_HUMAN]	2.52	1	1	1	2
Q96I59	Probable asparagine--tRNA ligase, mitochondrial OS=Homo sapiens GN=NARS2 PE=1 SV=3 - [SYNM_HUMAN]	2.52	2	1	1	2
Q8TB03	Uncharacterized protein CXorf38 OS=Homo sapiens GN=CXorf38 PE=1 SV=1 - [CX038_HUMAN]	2.51	1	1	1	4
P21912	Succinate dehydrogenase [ubiquinone] iron-sulfur subunit, mitochondrial OS=Homo sapiens GN=SDHB PE=1 SV=3 - [DHSB_HUMAN]	2.50	1	1	1	2
O96008	Mitochondrial import receptor subunit TOM40 homolog OS=Homo sapiens GN=TOMM40 PE=1 SV=1 - [TOM40_HUMAN]	2.49	1	1	1	1
Q13428	Treacle protein OS=Homo sapiens GN=TCOF1 PE=1 SV=3 - [TCOF_HUMAN]	2.42	2	3	3	4
Q8N4C8	Misshapen-like kinase 1 OS=Homo sapiens GN=MINK1 PE=1 SV=2 - [MINK1_HUMAN]	2.40	1	1	3	3
Q9UPY6	Wiskott-Aldrich syndrome protein family member 3 OS=Homo sapiens GN=WASF3 PE=2 SV=2 - [WASF3_HUMAN]	2.39	1	1	1	2
Q99717	Mothers against decapentaplegic homolog 5 OS=Homo sapiens GN=SMAD5 PE=1 SV=1 - [SMAD5_HUMAN]	2.37	1	1	1	1
Q9UKZ1	UPF0760 protein C2orf29 OS=Homo sapiens GN=C2orf29 PE=1 SV=1 - [CB029_HUMAN]	2.35	1	1	1	1
O43660	Pleiotropic regulator 1 OS=Homo sapiens GN=PLRG1 PE=1 SV=1 - [PLRG1_HUMAN]	2.33	1	1	1	2
O43818	U3 small nucleolar RNA-interacting protein 2 OS=Homo sapiens GN=RRP9 PE=1 SV=1 - [U3IP2_HUMAN]	2.32	1	1	1	1
Q01518	Adenylyl cyclase-associated protein 1 OS=Homo sapiens GN=CAP1 PE=1 SV=5 - [CAP1_HUMAN]	2.32	1	1	1	1
Q70UQ0	Inhibitor of nuclear factor kappa-B kinase-interacting protein OS=Homo sapiens GN=IKIP PE=1 SV=1 - [IKIP_HUMAN]	2.29	1	1	1	2
P61421	V-type proton ATPase subunit d 1 OS=Homo sapiens GN=ATP6V0D1 PE=1 SV=1 - [VA0D1_HUMAN]	2.28	1	1	1	2
Q9UPW0	Forkhead box protein J3 OS=Homo sapiens GN=FOXJ3 PE=1 SV=2 - [FOXJ3_HUMAN]	2.25	1	1	1	1
Q12805	EGF-containing fibulin-like extracellular matrix protein 1 OS=Homo sapiens GN=EFEMP1 PE=1 SV=2 - [FBLN3_HUMAN]	2.23	1	1	1	3
Q9BTV5	Fibronectin type III and SPRY domain-containing protein 1 OS=Homo sapiens GN=FSD1 PE=1 SV=1 - [FSD1_HUMAN]	2.22	1	1	1	1
O14874	[3-methyl-2-oxobutanoate dehydrogenase [lipoamide]] kinase, mitochondrial OS=Homo sapiens GN=BCKDK PE=1 SV=2 - [BCKD_HUMAN]	2.18	1	1	1	1
P57772	Selenocysteine-specific elongation factor OS=Homo sapiens GN=EEFSEC PE=1 SV=4 - [SELB_HUMAN]	2.18	1	1	1	2
Q96BH1	E3 ubiquitin-protein ligase RNF25 OS=Homo sapiens GN=RNF25 PE=1 SV=1 - [RNF25_HUMAN]	2.18	1	1	1	1

Q9ULX3	RNA-binding protein NOB1 OS=Homo sapiens GN=NOB1 PE=1 SV=1 - [NOB1_HUMAN]	2.18	1	1	1	1
Q8ND56	Protein LSM14 homolog A OS=Homo sapiens GN=LSM14A PE=1 SV=3 - [LS14A_HUMAN]	2.16	1	1	1	1
Q9UFC0	Leucine-rich repeat and WD repeat-containing protein 1 OS=Homo sapiens GN=LRWD1 PE=1 SV=2 - [LRWD1_HUMAN]	2.16	1	1	1	2
P10398	Serine/threonine-protein kinase A-Raf OS=Homo sapiens GN=ARAF PE=1 SV=2 - [ARAF_HUMAN]	2.15	1	1	1	1
P40692	DNA mismatch repair protein Mlh1 OS=Homo sapiens GN=MLH1 PE=1 SV=1 - [MLH1_HUMAN]	2.12	1	1	1	3
Q9BUK6	Protein misato homolog 1 OS=Homo sapiens GN=MSTO1 PE=1 SV=1 - [MSTO1_HUMAN]	2.11	1	1	1	2
Q10713	Mitochondrial-processing peptidase subunit alpha OS=Homo sapiens GN=PMPCA PE=1 SV=2 - [MPPA_HUMAN]	2.10	1	1	1	1
Q6DD88	Atlastin-3 OS=Homo sapiens GN=ATL3 PE=1 SV=1 - [ATLA3_HUMAN]	2.03	1	1	1	1
O43813	LanC-like protein 1 OS=Homo sapiens GN=LANCL1 PE=1 SV=1 - [LANC1_HUMAN]	2.01	1	1	1	1
Q6IBS0	Twinfilin-2 OS=Homo sapiens GN=TWF2 PE=1 SV=2 - [TWF2_HUMAN]	2.01	1	1	1	1

*****Total lysates of HUVECs monolayers were subjected to immunoprecipitation with anti-endoglin antibodies. Immunoprecipitates were analyzed by SDS-PAGE, followed by staining with Coomassie Brilliant Blue. Then, each lane of the gel was digested with trypsin and the resulting peptides were analyzed by mass spectrometry. The list of putative endoglin-interacting proteins was corrected by subtracting those proteins identified due to their interaction with beads alone or beads loaded with control IgG. Two hundred and forty eight proteins with a coverage score ≥2 are listed. Tripartite motif-containing protein 21 (TRIM21) unique peptide LGDTQQSIPGNEER was identified (row with yellow background) with a coverage percentage of protein of 2.95%. OS, Organism species; GN, Gene name; PE, Protein existence; SV, Sequence version.
